# Increasing prevalence of illicit drug use among employees at Swedish workplaces over a 25-year period

**DOI:** 10.1093/eurpub/ckac105

**Published:** 2022-08-25

**Authors:** Kristin Feltmann, Tomas Villén, Olof Beck, Johanna Gripenberg

**Affiliations:** STAD (Stockholm Prevents Alcohol and Drug Problems), Stockholm, Sweden; Department of Clinical Neuroscience, Centre for Psychiatry Research, Karolinska Institutet & Stockholm Health Care Services, Region Stockholm, Stockholm, Sweden; Department of Clinical Pharmacology, Karolinska University Laboratory, Stockholm, Sweden; Department of Clinical Neuroscience, Centre for Psychiatry Research, Karolinska Institutet & Stockholm Health Care Services, Region Stockholm, Stockholm, Sweden; STAD (Stockholm Prevents Alcohol and Drug Problems), Stockholm, Sweden; Department of Clinical Neuroscience, Centre for Psychiatry Research, Karolinska Institutet & Stockholm Health Care Services, Region Stockholm, Stockholm, Sweden

## Abstract

**Background:**

Reports indicate that the proportion of adults using drugs of abuse has been increasing in recent years in Europe. Although there are various indicators of increased drug use in Sweden over time, few studies could demonstrate an increase in the proportion of adults using drugs. To investigate changes in drug use prevalence over time, drug testing at the workplace has been used for a 25-year period.

**Methods:**

The urine samples of employees sent by occupational health services from all over Sweden during a 25-year period were analyzed. The analyzing capacity increased over time (from 3411 in 1994 to 60 315 samples analyzed in 2019), and the majority of the samples was analyzed for the following drugs: cannabis (tetrahydrocannabinol), amphetamine, opiates, cocaine, and benzodiazepines.

**Results:**

There was an overall increase in the proportion of samples that tested positive for illicit drugs over a 25-year period. This increase seemed to take place step-wise, with phases of linear increases and plateaus that over time became shorter. About 1.3% of samples tested positive for drugs in 1994, whereas 5.6% tested positive in 2019. Since 2007, the rate of positive samples has increased for cannabis and decreased for benzodiazepines. Although the rate of samples tested positive for opiates had remained relatively stable over the last 20 years, this rate had increased for amphetamine and cocaine between 2013 and 2019.

**Conclusion:**

The results indicate that the use of illicit drugs among employees at Swedish workplaces has increased during a 25-year period.

## Introduction

The use of psychoactive drugs can lead to significant acute adverse health effects, such as tachycardia, sudden elevated blood pressure, symptoms of anxiety and psychosis, and aggression.^1^ Chronic use of these substances can cause mental disorders, including physical and psychological dependence, such as substance use disorders.^1^^,^^2^ People suffering from substance use disorders have difficulties controlling their drug use, may neglect major roles and responsibilities and may have social and interpersonal problems, which can harm people close to them, mainly partners and children.^3^^,^^4^ Because of these acute and chronic effects, drug use causes a significant health burden for society.^5^^,^^6^ Degenhardt and Hall stated that better data about prevalence of illicit drug use was needed in order to guide appropriate policy responses.^6^

Many different types of illicit drugs are used for recreational purposes. Cannabis is the most widely used illicit drug worldwide and has been associated with psychosis, anxiety, depression and cognitive deficits.^6–11^ Although the use of cannabis is illegal in most countries, it has been legalised in Canada and several states in the USA, among others. The central stimulants amphetamines and cocaine are popular party drugs.^12–16^ However, the use of amphetamines and prescription stimulants is also common among college students in the USA, aiming to enhance academic performance.^17^^,^^18^ Opioids are potent pain medications with high abuse potential. With chronic use, tolerance is induced; that is, higher doses are needed to obtain the same psychoactive effect. However, high doses of opioids can cause life-threatening respiratory depression. For several decades, the number of opioid users and fatal opioid overdoses in the USA has been consistently rising, causing a widespread public health crisis.^19^^,^^20^

Both in Europe overall and Sweden specifically, drug use was highest among young adults, and cannabis was the most commonly used drug of abuse.^21^^,^^22^ The estimated prevalence of cannabis use among young adults has increased over time in countries that initially had a lower prevalence^21^ and in Sweden.^22^ In 2017, 4% of people aged 17–84 years in Sweden reported using at least one substance classified as a narcotic in the previous 12 months.^22^^,^^23^ Between 2013 and 2017, the estimated prevalence of cannabis, cocaine and ecstasy use increased.^23^ Other indicators of increased drug use over time were increases in the number of drug confiscations, convictions for drug-related crimes, treatments for substance use disorders and drug-related deaths.^24^ However, according to the report, ‘sporadic or recreational illicit drug use, [did] not show any major signs of increase’ and, therefore, the authors concluded that the proportion of people using drugs has not increased but that among users, drug use frequency had increased.^24^

Understanding drug trends over time can help guide appropriate responses, including preventive, corrective, and treatment efforts. If drug use is increasing over time and not just fluctuating, there might be a need to provide more resources to municipalities, counties, the police, and the health care system to both reduce drug availability and use and to treat people with substance use disorder early. These measures could avoid individual suffering and reduce societal costs related to long-term drug use. In addition, the demand for drugs is thought to finance gang-related violence, which has increased in Sweden.^25^^,^^26^ Therefore, reducing demand might hopefully also decrease competition in the market and related violence.

Drug testing at the workplace has been established for a long time in Sweden and comprises pre-employment and random testing and can be used as an indicator of drug use prevalence over time. As a refusal of drug testing is reported to the employer, drug testing at the workplace is likely to be associated with fewer dropouts and selection bias than studies using self-reported surveys. Furthermore, unlike nightlife scenes where drug use is high,^12^^,^^13^^,^^27^ workplaces are not typical environments for drug use. Therefore, drug testing in these environments might be a better indicator of drug use prevalence among the general adult population. Drug testing of individuals is also more suitable to study changes in prevalence over time, as self-reported data can be influenced by changes in social acceptance of a drug or survey methodology.^28^^,^^29^

The current study investigated changes in the proportion of adult employees in Sweden who had tested positive for an illicit or prescription drug over a 25-year period. Employees who have been subjected to drug testing at their workplaces delivered biological samples to occupational health services. These samples were immediately analyzed for the use of illicit drugs and addictive prescription drugs by forensic standard drug testing methods.

## Methods

### Selection

All urine specimens coming to the laboratory were analyzed according to requests. The samples were from Swedish workplaces that had implemented pre-employment and random drug testing as part of a drug policy program. Sampling was conducted at the local occupational health unit and followed routines mandated in the accreditation of the laboratory. Routines were according to the European Workplace Drug Testing Society (EWDTS) guidelines and the College of American Pathologists (CAP) forensic drug testing requirements. All positive results were reported to the medical review officer (MRO) for further processing. In the case of challenged test results, the stored B sample was sent to another laboratory for analysis. No personal data was collected for the study.

### Chemical analysis

Drug screening was performed using standard immunochemical reagents following the cut-off level and calibrators recommended by the manufacturer. Confirmations of positive screening results were done by mass spectrometry methods. All positive results used for presenting the data in this report were according to forensic standards. No important changes in methodology have been made over the years. The detection time was estimated to be between 1 and 4 days for different drugs, depending on many factors.^29^

### Ethical considerations

Analytical investigations were always according to request. No information regarding individual or workplace identity was recorded. Drug testing at workplaces is intended as a preventive measure. Workplaces using drug testing have a drug policy stating that employees are expected to be drug and alcohol-free at work and that random drug screening can take place, which is meant to discourage drug use. In the event of a positive drug test, results are handled within a medical context to see if the individual is suffering from a substance use disorder and is in need of treatment. Drug tests can be used to monitor if employees remain drug-free. The content and formulation of these policies vary between workplaces, and how drug-positive tests are treated depends on each individual case, and data on this in the current study are lacking. Since the consequences for employees can be severe, drug testing followed the EWDTS and CAP standards mentioned above. According to these guidelines, sampling was conducted by professionals at the occupational health services who followed certain routines and performed checks; the analyzing laboratory was accredited, and an MRO handled positive results and implemented quality assurance of drug testing. In the case that a positive test result was disputed, the b-sample was analyzed in another laboratory.

### Statistical analysis

**Figure 1:**
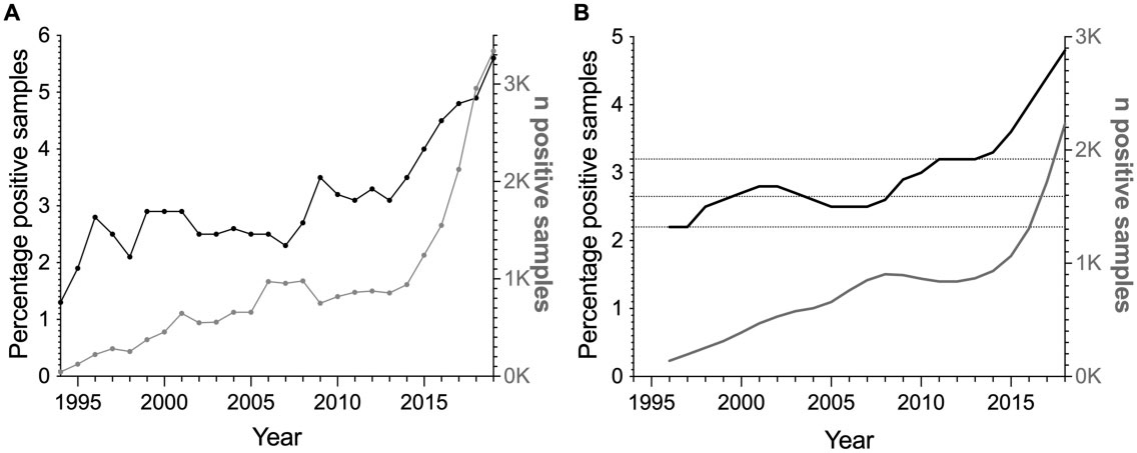
Samples tested positive for drugs of abuse over time. (A) The number of samples tested positive for any drug of abuse screened for are presented in total number (grey dots and line, K for 1000) and as percentages of all samples screened (black dots and lines). These values represent the sum of the analyses performed each year. (B) To smooth the data and observe potential patterns, the data points in A have been transformed to a moving mean of 4 years and a centred mean of two moving means. The dotted lines represent new plateaus where the percentage of positive samples has increased over time

Positive samples are presented as the number and percentage of samples analyzed from 1994 (3411 samples) to 2019 (60315 samples). To investigate changes over time in the percentage of samples tested positive for different drugs, data were analyzed from 1998 and onwards (since in 1998, at least 1000 samples were screened for each drug and based on the assumption that the yearly prevalence can be more reliably assessed with a larger number of tests). To smooth the data (figure 1B), the moving mean of 4 years and the centred mean of two moving means were created. Linear regression was performed to analyze periods of linear increases in the proportion of positive samples.

## Results

### Overall drug use

The total and percentage of all analyzed samples that tested positive for drugs of abuse (illicit drugs and prescription medications; table 1) has increased in the 25-year period (figure 1A). In 1994, 1.3% of samples tested positive, whereas, in 2019, 5.6% tested positive, which equals an increase of 315%. The average percentage change from 1 year to the next is +7%.

**Table 1:** Overview of drugs analyzed

Drug classes (targets)	Drugs tested
Cannabinoids	Tetrahydrocannabinol (THC)^a^, synthetic cannabinoid (‘spice’)
Central stimulants	Cocaine^a^, amphetamine^a^
Opioids	Opiates (morphine, heroin)^a^, oxycodone, tramadol, dextro-propoxyphene, ketobemidone, buprenorphine, methadone
Sedatives	Benzodiazepines^a, b^ barbiturates, zolpidem, zopiclone
Hallucinogens	Psilocybin (Mushrooms), lysergic acid diethylamide (LSD)
Others	Phencyclidine (PCP), gamma-hydroxybutyric acid (GHB)

The various drugs of abuse that have been analyzed in the present study and the respective drug classes are presented. Common to these drugs is that they are addictive, i.e. can produce physical and psychological dependence.

a Drugs that have been screened for in the majority of the samples.

Smoothing of the data reveals a pattern of a step-wise increase in the percentage of positive samples between 1994 and 2019. There are three periods of increase connected by two phases of plateaus (figure 1B). The first plateau is characterised by slight increases and decreases around a certain percentage. The second plateau lasted for a shorter time (2011–13) than the first one (2000–08). Between 2013 and 2019, the percentage of positive drug samples increased linearly by about 0.40% yearly (−810.70 + 0.40×Year, Adjusted *R*^2^=0.97; *F*_1,6_=190.33, *P* < 0.001).

### Use of different substances

**Figure 2:**
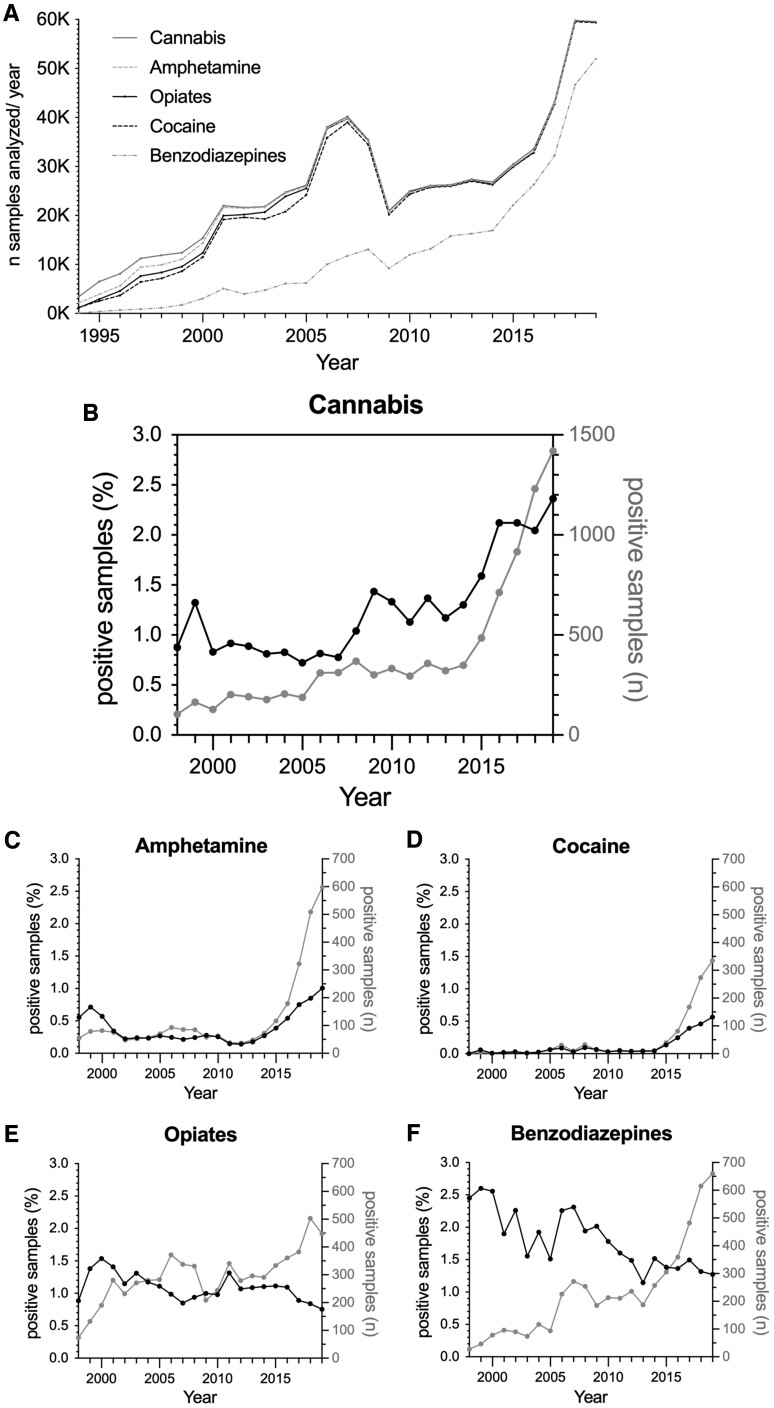
Changes in the detection rate of drugs of abuse most commonly tested. (A) The number of analyses per year performed for each drug is presented. (B–F) The data present the samples that tested positive for a certain drug in total numbers (grey-dotted line) and as percentages of all samples analyzed for this drug (black-dotted line)

There are changes over time in the proportion of positive tests regarding the most frequently analyzed drugs: cannabis, opiates, amphetamine, cocaine and benzodiazepines (figure 2). Overall, the average yearly percentage of positive samples for the period 1998–2019 was highest for benzodiazepine-screened samples (mean±SEM: 1.80 ± 0.09%), followed by cannabis (mean±SEM: 1.26 ± 0.11%) and opiates (mean±SEM: 1.09 ± 0.04%), and lowest for amphetamine (mean±SEM: 0.39 ± 0.05%) and cocaine (mean±SEM: 0.11 ± 0.03%). However, in 2019, cannabis is the most commonly detected drug (2.36%), followed by benzodiazepines (1.27%) and amphetamine (1.00%).

During the period analyzed, an increase in the proportion of positive samples can be observed for cannabis (2007–19, 0.78–2.36%, figure 2B) and, more recently, for amphetamine (2012–19, 0.14–1.00%, figure 2C) and cocaine (2014–19, 0.04–0.56%, figure 2D). In contrast, the number of samples positive for opiates has remained relatively stable (1998–2019, 0.89–0.75%, figure 2E). Although the proportion of samples tested positive for benzodiazepines fluctuated during the first years, it decreased between 2007 and 2019 (2.31–1.27%, figure 2F).

Linear regression was performed for various drugs during the period between 2013 and 2019 when approximately linear increases and decreases could be observed. Increases in positive samples were greatest for cannabis (−399.95 + 0.20×Year, Adjusted *R*^2^=0.85; *F*_1,6_=36.21, *P* = 0.002), followed by amphetamine (−285.99 + 0.14×Year, Adjusted *R*^2^=0.99; *F*_1,6_=528.01, *P* < 0.001) and cocaine (−191.25 + 0.10×Year, Adjusted *R*^2^=0.97; *F*_1,6_=165.32, *P* < 0.001), and were absent for opiates (127.70 + 0.06×Year, Adjusted *R*^2^=0.78; *F*_1,6_=21.73, *P* = 0.006) and benzodiazepines (−3.68 + 0.003×Year, Adjusted *R*^2^=−0.20; *F*_1,6_=0.01, *P* = 0.929) between 2013 and 2019.

Since other drugs were less routinely requested in the analysis, few trends regarding the proportion of positive tests can be analyzed (see table 1). However, the number of samples screened for these drugs, as well as the number of samples that tested positive, can be found in the Supplementary data. For example, test results can be found for anabolic–androgenic steroids (Supplementary table S1), opioids like methadone (Supplementary table S2) and tramadol (Supplementary table S3), as well as sedatives like Z-drugs (Supplementary table S3).

### Polydrug use

In general, in the majority of the positive samples during the period of 1998–2018, one type of drug was detected (mean±SEM over 1998–2018: 88.99 ± 0.62%). However, the percentage of the positive samples in which two and three types of drugs were detected was 8.97 ± 0.47% and 1.66 ± 0.18% (mean±SEM over 1998–2018), respectively.

**Figure 3:**
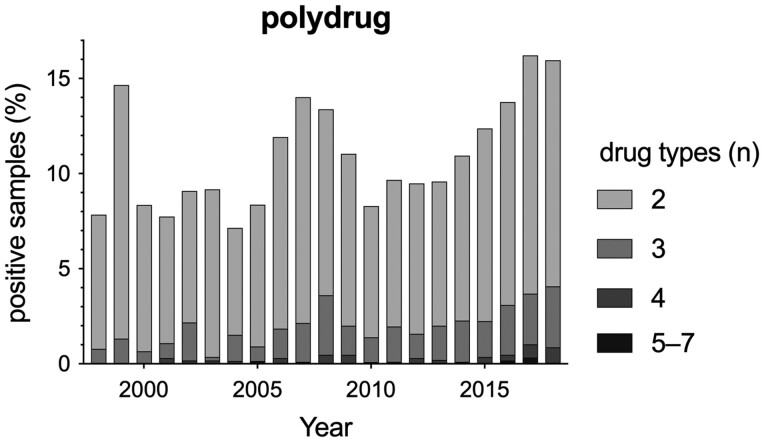
Number of drugs identified per analyzed sample. Samples positively tested for more than one drug type are presented as percentages of all samples positively tested during that year. Percentages have been stacked to identify the extent of polydrug use.

Nevertheless, the number of samples that tested positive for two or three drugs increased from 7.06% to 11.89% (68% increase) and 0.78% to 3.20% (310% increase) between 1998 and 2018, respectively (figure 3). Furthermore, the percentage of samples that tested positive for two or more types of drugs increased linearly between 2013 and 2018 (−2814.86 + 1.40×Year, Adjusted *R*^2^=0.94; *F*_1,5_=85.68, *P* = 0.001).

## Discussion

In the present study, we demonstrated that over a 25-year period (1994–2019), an increasing number of adults were positive for illicit drugs when tested in the workplace. In recent years (2013–19), there were linear increases in the proportion of samples that tested positive for cannabis, cocaine and amphetamine, but no changes were observed for opiates and benzodiazepines. In addition, the proportion of positive samples for more than one drug also increased between 2013 and 2018. Therefore, the study indicates that the use of illicit drugs has been increasing in recent years.

During the 25-year period, the proportion of samples being tested positive for one or more drugs of abuse has increased. These results might reflect that the drug use prevalence, that is, the proportion of adults who use drugs, is increasing. Moreover, an increasing frequency of drug use could also have led to a higher proportion of positive tests, as the risk of being detected is higher with increased use. The present study is in line with previous reports suggesting an increase in drug use in Europe and Sweden.^21^^,^^23^^,^^24^^,^^30^

The proportion of samples that tested positive for cannabis in the present study increased between 2007 and 2019. In the last year included in the study, cannabis was the most commonly detected drug. Reports based on population-wide surveys confirmed cannabis as the most widely used illicit drug and reported an increase in the 12-month use prevalence during recent years in Sweden.^22–24^ Furthermore, the number of first-time entrants to treatment for cannabis use has been increasing since 2011 in Sweden.^22^ Nevertheless, self-reported cannabis use was lower in Sweden than in other European countries.^21^ In recent years, cannabis has been legalised in several countries, and the attitude towards cannabis and the perceived risk associated with its use has changed,^31–34^ which might have contributed to an increased acceptance of the drug and higher levels of use.

Cocaine and amphetamine were less often detected than cannabis. However, the proportion of positive samples has increased for both drugs since about 2013. These increases are in line with studies from Sweden and Europe. In Sweden, the estimated prevalence of cocaine use was higher in 2017 than in 2013,^23^ and the number of people searching for treatment for cocaine problems for the first time increased between 2011 and 2017.^22^ In Europe, an increase in cocaine use was also indicated by the increasing levels of cocaine metabolites in wastewater in European cities in recent years.^30^ Similarly, the number of first-time entrants for amphetamine treatment in Europe,^21^ as well as the levels of amphetamine metabolites in wastewater in Finland, had increased.^30^ A recent study confirmed that levels of amphetamine and cocaine metabolites measured in Stockholm were similar to the other Nordic capitals.^35^ Together, the present study’s data and previous reports indicate that cocaine and amphetamine use has increased in recent years.

In the present study, the proportion of people who tested positive for opiates has remained relatively stable over the years. Opiate findings in workplace samples were predominantly related to codeine and morphine intake. These findings are in line with a recent study showing that opioid prescriptions (which included opiates) did not change between 2006 and 2014 in Sweden and that the relative contribution of morphine and codeine to prescribed opioid use was similar between 2006 and 2014.^36^ A study in the USA has shown that opioid-related deaths have been rising exponentially during the last decades, suggesting that the current opioid epidemic might not only be attributed to a surge of prescriptions in the 1990s but also the increased availability due to technological advances (supply), as well as social and psychological problems (increased demand).^20^ In Europe, although the consumption of prescribed opioids increased between 2004 and 2016, there is currently no evidence of an opioid crisis.^37^ Nevertheless, the mortality rate among young adults between 2000 and 2017 had not decreased in Sweden as much as in the rest of Europe, which has mainly been related to opioid use.^38^

For many years, benzodiazepines remained the most detected type of psychoactive drug, despite an overall decline in detection rate over time. Benzodiazepines are prescribed for anxiety, insomnia, and seizures, and their legal status is likely related to the relatively high prevalence. However, long-term use can induce dependence, and new benzodiazepines are constantly appearing on the illegal market, indicating a high demand.^21^ Hence, the use of other benzodiazepines than the ones tested could further contribute to the actual prevalence and might counteract the observed decline in the present study.

Several factors could have contributed to increased illicit drug use in recent years. The first increase in drugs (after smoothing: from 1994 to 2001) found in the present sample coincides with a decrease in street prices in Sweden for the following drugs: cannabis, heroin, amphetamines, and cocaine.^24^ After this period, prices remained rather stable until 2015, except for a slight increase in prices for cannabis, which occurred while tetrahydrocannabinol levels were rising simultaneously (see Ref.24, table 38). The increasing number of amphetamine and cocaine seizures (see Ref.24, tables 33–36), together with the absence of a rise in street prices, could indicate the increased availability of drugs. The European Monitoring Centre for Drugs and Drug Addiction (EMCDDA) report from 2019 also states that in Europe, ‘cocaine availability is at an all-time high’.^21^ Furthermore, improved communications technology during the previous two decades could have also improved supply chains and increased drug availability.^39^ According to previous studies, changes in drug availability influence drug use,^40^ and the present study supports this notion. Moreover, several other psychological and sociological factors could have increased drug use, such as changes in risk perceptions, attitudes and norms; economic insecurity; and a rise in mental health problems.^20^^,^^31^^,^^32^^,^^38^

The present study indicates that the prevalence of cannabis use might have increased since 2007 and that the prevalence of cocaine and amphetamine use might have increased since 2013. For the year 2017, the proportion of positive test results for all these drugs is higher than the proportion of respondents in a Swedish national survey stating their last 30-day use.^23^ This is counterintuitive since drug testing detects drug use during the previous 4 days only and, therefore, should be lower. Similarly, the report states that in 2017, 4% of people between 17 and 84 reported having used at least one drug classified as a narcotic during the previous 12 months. In the present study, in 2017, 4.8% of drug tests returned positive for a drug classified as a narcotic. The Swedish report, whose data are also used for the estimated prevalence in Sweden in the EMCDDA reports,^22^ states that it had a dropout rate of 40% and that men and young people are under-represented. Hence, in light of the current study results, the previous survey data might have under-estimated drug use among the general adult population. Considering the significant acute and chronic negative health effects of drugs, the current study indicating an increasing use of illicit drugs and relatively high use of prescription drugs should motivate authorities to increase prevention and treatment efforts in Sweden.

### Limitations and strengths

The present study has certain limitations. First, it contains no information on individuals’ time of employment (e.g. newly employed), history (tested previously), age, sex, or profession. Second, due to the detection time of up to 4 days, the drug use prevalence in the population (considering a longer period) could be higher. Third, the drug use prevalence could also be higher among the working-age population due to a potential selection bias created by companies that choose to drug test their employees and inform them about such testing. Nevertheless, the study’s strength is the large number of individuals tested. Considering that in 2019, about 5 million Swedish people were working and 60 000 were subjected to drug testing, around 1.2% of the entire working population was screened. Furthermore, there is less risk of a selection bias since testing is less voluntary, as even a refusal to be tested is reported back to the employer. However, this raises ethical concerns as even non-problematic drug use is not accepted and is potentially stigmatised.

## Funding

The authors received no financial support for the research, authorship, and/or publication of this article.

## Supplementary data

Supplementary data are available at *EURPUB* online.

*Conflicts of interest*: None declared.

Key points

- The proportion of samples that tested positive for illicit drugs increased between 1994 and 2019.
- In recent years, the proportion of samples that tested positive for cannabis, amphetamine, and cocaine increased.
- Benzodiazepines and opiates are prevalent but indicate no increase over time.
- Over time, an increasing percentage of samples have been tested positively for more than one type of drug, indicating polydrug use.
- This indicated increase in drug use calls for preventive interventions to reduce the public health burden.

## Supplementary Material

ckac105_Supplementary_Data

## Data Availability

The data underlying this article will be shared upon reasonable request to the corresponding author.
