# Supplementary material for: Increasing prevalence of illicit drug use among employees at Swedish workplaces over a 25-year period
Source: Eur J Public Health. 2022 Aug 25;32(5):760–5. doi: 10.1093/eurpub/ckac105 (PMC9527979; doi:10.1093/eurpub/ckac105)
Supplement: ckac105_Supplementary_Data [file ckac105_supplementary_data.docx]

**Supplementary Information**

A number of drugs were tested at a much lower frequency than the drugs mentioned above (Tables S1-S4). It can be assumed that these tests might be to a lower extent random and therefore less representative of the working adult population.

The opioid dextropropoxyphene was detected to a certain extent when considering years of at least 1000 analyzed samples, usually between 0 and 1% (2001-2012) and 0% after 2012 (Table S1), confirming a study showing that prescriptions had stopped in Scandinavian countries (1). The average yearly percentage of positive tests for the doping drugs anabolic-androgenic steroids was 1.86±0.13% (mean±SEM) for the period 1994-2019. There is no apparent overall increase or decrease in the percentage of positive samples for these drugs (Table S1).

For the drugs where the number of analyzed samples was low, no percentages were calculated (Tables S2-S4). However, some observations can be made. The hallucinogens lysergic acid diethylamide (LSD) and psilocybin, as well as phenylcyclohexyl piperidine (PCP), were rarely or never detected. Other opioids, such as methadone and buprenorphine (substitution treatments), tramadol and oxycodone (prescription drugs) were screened for to a lesser extent but were detected in around 1 to 10%. Regarding hypnotics, barbiturates and gamma-Hydroxybutyric acid (GHB) were rarely detected but almost every year there were positive tests for zolpidem and zolpiclone.

Few screens have been performed for Spice (synthetic cannabinoid) but in recent years there were positive results every year.

**Table S1.**  **Number of positive samples tested for drugs of abuse.**

The number of samples analyzed for drugs of abuse (n) and the number of samples tested positive (+), as well as the percentage of positive samples (%) for these drugs are presented.

|  | **Dextropropoxyphene** | | | **Anabolic-Androgenic Steroids** | | |
| --- | --- | --- | --- | --- | --- | --- |
|  | n | + | % | n | + | % |
| **1994** | 1 | 0 | 0 | 31 | 1 | 3.2 |
| **1995** | 2 | 0 | 0 | 274 | 1 | 0.4 |
| **1996** | 23 | 0 | 0 | 172 | 5 | 2.9 |
| **1997** | 66 | 1 | 1.5 | 486 | 6 | 1.2 |
| **1998** | 68 | 0 | 0 | 694 | 14 | 2.0 |
| **1999** | 87 | 1 | 1.1 | 442 | 3 | 0.7 |
| **2000** | 141 | 5 | 3.5 | 535 | 10 | 1.9 |
| **2001** | 773 | 6 | 0.8 | 1126 | 21 | 1.9 |
| **2002** | 2714 | 21 | 0.8 | 972 | 25 | 2.6 |
| **2003** | 2307 | 18 | 0.8 | 1243 | 20 | 1.6 |
| **2004** | 2455 | 15 | 0.6 | 973 | 24 | 2.5 |
| **2005** | 2128 | 15 | 0.7 | 1087 | 27 | 2.5 |
| **2006** | 3054 | 23 | 0.8 | 1573 | 16 | 1.0 |
| **2007** | 4152 | 42 | 1.0 | 1824 | 32 | 1.8 |
| **2008** | 5378 | 16 | 0.3 | 1307 | 20 | 1.5 |
| **2009** | 5604 | 23 | 0.4 | 690 | 11 | 1.6 |
| **2010** | 4118 | 25 | 0.6 | 808 | 11 | 1.4 |
| **2011** | 4595 | 16 | 0.3 | 813 | 20 | 2.5 |
| **2012** | 3662 | 1 | 0 | 685 | 14 | 2.0 |
| **2013** | 3460 | 0 | 0 | 631 | 11 | 1.7 |
| **2014** | 2643 | 0 | 0 | 664 | 7 | 1.1 |
| **2015** | 1275 | 0 | 0 | 1051 | 29 | 2.8 |
| **2016** | 1082 | 0 | 0 | 936 | 10 | 1.1 |
| **2017** | 971 | 0 | 0 | 960 | 17 | 1.8 |
| **2018** | 1080 | 0 | 0 | 1178 | 31 | 2.6 |

|  | **Methadone** | | **Barbiturates** | | **PCP** | | **LSD** | | **GHB** | |
| --- | --- | --- | --- | --- | --- | --- | --- | --- | --- | --- |
|  | n | + | n | + | n | + | n | + | n | + |
| **1993** | 5 | 0 | 5 | 0 | 1 | 0 |  |  |  |  |
| **1994** | 10 | 3 | 4 | 0 | 1 | 0 |  |  |  |  |
| **1995** | 20 | 0 | 27 | 1 | 33 | 0 |  |  |  |  |
| **1996** | 19 | 0 | 49 | 0 | 3 | 0 |  |  |  |  |
| **1997** | 90 | 0 | 139 | 0 | 13 | 0 |  |  |  |  |
| **1998** | 86 | 0 | 85 | 0 | 14 | 0 | 879 | 0 |  |  |
| **1999** | 62 | 0 | 64 | 0 | 17 | 0 | 964 | 0 |  |  |
| **2000** | 50 | 0 | 72 | 0 | 12 | 0 | 1638 | 0 |  |  |
| **2001** | 31 | 0 | 261 | 0 | 8 | 0 | 1788 | 0 | 174 | 11 |
| **2002** | 47 | 0 | 62 | 0 | 60 | 0 | 2434 | 0 | 67 | 0 |
| **2003** | 26 | 0 | 26 | 0 | 8 | 0 | 1607 | 0 | 51 | 0 |
| **2004** | 32 | 0 | 34 | 0 | 9 | 0 | 1640 | 0 | 35 | 0 |
| **2005** | 44 | 0 | 64 | 0 | 12 | 0 | 1972 | 0 | 115 | 0 |
| **2006** | 23 | 1 | 43 | 0 | 446 | 0 | 1694 | 0 | 235 | 4 |
| **2007** | 18 | 2 | 48 | 0 | 847 | 0 | 1705 | 6 | 261 | 2 |
| **2008** | 136 | 0 | 25 | 0 | 958 | 0 | 1188 | 4 | 271 | 1 |
| **2009** | 281 | 1 | 31 | 0 | 702 | 0 | 504 | 2 | 144 | 0 |
| **2010** | 36 | 0 | 33 | 0 | 282 | 0 | 22 | 1 | 48 | 0 |
| **2011** | 23 | 2 | 21 | 0 | 188 | 0 | 57 | 0 | 40 | 0 |
| **2012** | 112 | 1 | 8 | 0 | 222 | 0 | 61 | 0 | 19 | 0 |
| **2013** | 175 | 1 | 107 | 0 | 187 | 0 | 21 | 0 | 39 | 0 |
| **2014** | 261 | 2 | 230 | 0 | 400 | 0 | 12 | 0 | 26 | 0 |
| **2015** | 358 | 1 | 294 | 0 | 388 | 0 | 12 | 0 | 30 | 0 |
| **2016** | 358 | 1 | 272 | 0 | 310 | 0 | 9 | 0 | 23 | 0 |
| **2017** | 357 | 3 | 302 | 0 | 350 | 0 | 44 | 3 | 23 | 0 |
| **2018** | 488 | 4 | 332 | 0 | 344 | 0 | 48 | 0 | 12 | 0 |

**Table S2.**  **Number of positive samples tested for drugs of abuse.** The number of samples analyzed for drugs of abuse (n) and the number of samples tested positive (+) for these drugs are presented.

**Table S3.** **Number of positive samples tested for drugs of abuse.** The number of samples analyzed for drugs of abuse (n) and the number of samples tested positive (+) for these drugs are presented.

|  | **Tramadol** | | **Ketobemidone** | | **Buprenorphine** | | **Zolpidem** | | **Zopiclone** | |
| --- | --- | --- | --- | --- | --- | --- | --- | --- | --- | --- |
|  | n | + | n | + | n | + | n | + | n | + |
| **2006** | 212 | 2 | 3 | 0 | 160 | 0 | 239 | 0 | 226 | 2 |
| **2007** | 302 | 14 | 0 | 0 | 295 | 9 | 290 | 1 | 303 | 25 |
| **2008** | 310 | 24 | 3 | 0 | 252 | 7 | 250 | 11 | 232 | 8 |
| **2009** | 246 | 8 | 0 | 0 | 610 | 5 | 240 | 10 | 252 | 29 |
| **2010** | 390 | 12 | 0 | 0 | 454 | 9 | 298 | 4 | 289 | 3 |
| **2011** | 448 | 19 | 8 | 1 | 221 | 10 | 307 | 18 | 290 | 7 |
| **2012** | 396 | 19 | 11 | 0 | 175 | 5 | 116 | 7 | 139 | 16 |
| **2013** | 835 | 40 | 44 | 0 | 223 | 4 | 236 | 8 | 208 | 30 |
| **2014** | 603 | 26 | 46 | 0 | 223 | 17 | 158 | 7 | 271 | 11 |
| **2015** | 835 | 28 | 33 | 3 | 382 | 19 | 417 | 24 | 578 | 33 |
| **2016** | 1560 | 59 | 14 | 1 | 485 | 13 | 236 | 11 | 282 | 12 |
| **2017** | 1479 | 66 | 0 | 0 | 402 | 17 | 0 | 0 | 0 | 0 |
| **2018** | 2316 | 100 | 0 | 0 | 520 | 8 | 0 | 0 | 0 | 0 |

**Table S4. Number of positive samples tested for drugs of abuse.** The number of samples analyzed for drugs of abuse (n) and the number of samples tested positive (+) for these drugs are presented.

|  | **Spice** | | **Oxycodone** | | **Psilocybin** | |
| --- | --- | --- | --- | --- | --- | --- |
|  | n | + | n | + | n | + |
| **2010** |  |  | 4 | 0 |  |  |
| **2011** | 12 | 0 | 2 | 0 | 3 | 0 |
| **2012** | 14 | 0 | 15 | 2 | 4 | 0 |
| **2013** | 948 | 7 | 65 | 0 | 17 | 0 |
| **2014** | 1011 | 11 | 73 | 3 | 6 | 0 |
| **2015** | 1038 | 10 | 85 | 2 | 15 | 0 |
| **2016** | 694 | 3 | 98 | 3 | 28 | 0 |
| **2017** | 343 | 2 | 231 | 10 | 35 | 0 |
| **2018** | 332 | 8 | 244 | 4 | 44 | 0 |

Bibliography

1. Jarlbaek L. Opioid prescribing habits differ between Denmark, Sweden and Norway - and they change over time. Scand J Pain. 2019 Jul 26;19(3):491–9.
